# 

**Published:** 1891-01

**Authors:** 


### INDEX TO VOL. XXII.

### ORIGINAL COMMUNICATIONS.

PAGE.
Abscesses, Phlegmonous...........76
Abdominal Surgery, Some Practical Points on. ............ ...... ..338
Administration of One-Twenty-fourth of a Grain of Merck's Hyoscyamine, Alarming Symptoms following the,............ .... 485
Bone Packing, five cases of—Sennis' Method... .................109
Bacteriology and its Relation to Scientific Medicine, Some Remarks on....................... ..168
Chloroform, Physiological action of.. ........................... 17
Continued fevers of the South, a contribution to the study of the..59
Consumption................ ....112
Constipation and its relation to Pelvci diseases in young women....219
Carbuncular Anthrax, treatment with report of cases............232
Clinical cases, interesting.........313
Chloralamid in surgery...........385
Circumcision without removing any part of prepuce and under the use of cocaine..............438
Children, diseases of. .......... ..478
Cancer of the uterus and its rational treatment.....................575
Children, diseases of... ...........368
Death after a dose of Salol.......350
Diphtheria.................... 177
Elbow-Joint, Fracture of, with Cases.......... ............... .. 12
Electricity, under what Conditions can it be of Positive Service to the Gynæcologist?..................118
Electric Freak, An.................371
Empyema.........................371
Extirpation of the Cancerous Uterus, is it a Justifiable Operation ?.434
Ergot, an Experimental and Clinical Study of......................439
Epithelioma, the Early Diagnosis of........................ .... 521
Faridization in Incontinence of Urine.......... ..............356
Gonorrhœa, Relation of the Physician to the Results of...........526

PAGE.
Hypertrophic Nasal Catarrh, Some of the Results of................165
Hæmoglobinuria, Malarial 365 427 538
Hiccough, the Treatment of......456
LaGrippe..........................77
LaGrippe, its Etiology, Clinical History and Treatment.........469
Laparotomy, Report of Ten Cases of............................... 591
LaGrippe, Its Treatment in Our City.............................49
Miscarriage, Retained Placenta in—How Shall We Treat Such Cases?
.................................381
Nitro-Glycerine and Nitrate of Amyl, The Therapeutic Uses of..531
New Obstetric Instrument, The...540
Œdema of the Glottis Following Influenza..........................340
Pneumonia in Childhood, Treatment of......................... 122
Pneumonia, Treatment of .......125
Pulmonary Phthisis, The Treatment of...................... 263 323
Philosophy, Medical..............373
Rib, Fracture of, Followed by Extensive Tissue Empyema.........7
Solid Ovarian Tumor, Removal of, with Grave Complications......213
Successful Laparotomy for Intussusception of the Bowels........281
Sciatica, a Case of, Terminating in Death.......... ...............319
Summer Diarrhœa in Children, Its Etiology and Rational Treatment
.................................583
Tonsils, Hypertrophy of the........1
Testimony, Expert.................67
Typho-Malarial Fever.... ........174
Threatened Puerperal Convulsions, When Should We Interfere in...274
Tuberculin, Is it a Failure?......417
Typhoid-Fever, Treatment of.....482
Urethral Irrigator and Irrigating Dilators, Lindenschmidt's......126
Uterine Ligaments, Complete Relaxation of all the...............370
Venereal Diseases, The Treatment of, in Charity Hospital..... ....159

### CORRESPONDENCE.

PAGE.
New York Letter 20, 79, 183, 236, 299, 388, 444, 494, 544, 603.
Vienna Letter.................357, 398
Paris Letter......................487
Berlin Letter... ...................596
A Much Needed Work............127

### SOCIETY NOTES.

Atlanta Society of Medicine 36, 187, 502.
Academy of Medicine and Surgery, Proceedings of the Richmond, Va ..............24, 128
American Medical Association....283
American Gynæcological Society..451
American Medical Association, Membership in the..............452
Chicago Pathological Society.....140
Gynæcological and Obstetrical Society of Baltimore. 133, 286, 347, 391
Medical Association of Georgia...240
Mississippi Valley Medical Association.....................451, 559
North Carolina State Medical Society.......................250, 341
Southern Surgical and Gynæcological Association.................607
Tennessee State Medical Society..243 294.
Tri-State Medical Society of Alabama, Georgia and Tennessee, The Third Annual Meeting of the.........................452, 547

### BOOK REVIEWS.

A Manual of Auscultation and Percussion, Flint....................31
A Manual of the Practice of Medicine, Taylor ......................84
A Guide to the Practical Examination of Urine, Tyson............179
Auscultation and Percussion, Shattuck............................197
A Compend of Diseases of Children, Hulfield .........................197
A Compend of Gynæcology, Morris, .............................252
A Treatise on the Diseases of the Nervous System, Hammond....503
Artificial Anæsthesia and Anæsthetics, Willard.................561
Comparative Physiology, Mills....29
Diseases of the Eye, Kettleship....83
Diseases of the Digestive Organs of Infancy and Childhood, Starr...447
Encyclopædia of the Diseases of Children, Keating...............147
Essentials of Minor Surgery and Bandaging, With an Appendix on Venereal Diseases, Martin......197
Equine Anat. and Phys., Ballon..306
Fever, Its Pathology and Treatment by Antipyretics, Hare..........396
Heath, Heredity and Personal Beauty, Shoemaker..................251
International Clinics.............560
International Clinics...............306
J. B. Lippincott Co...............252
Koch's Remedy, in Relation to Throat Consumption ...........307
Medical Diagnosis with Special Reference to Practical Medicine, DaCosta.........................32
Materia Medica, Pharmacology and Therapeutics, Shoemaker.......447
Medical Symbolism Illustrated, Sozinskey...........................503
Muter's Analytical Chemistry, Muter,.........................560
Modern Method of Antiseptic Wound Treatment..............619
Ointments and Oleates, Shoemaker,30
Practical Treatise on Impotency, Sterility and Allied Disorders of the Male Sexual Organs, Goss...85
Prof. Robt. Koch's Method to Cure Tuberculosis ...................85
Practical Notes on Urinary Analysis, Canfield.......................309
Principles of Surgery, Senn.......354
Practical Points in the Management of the Diseases of Children, Love,396
Plain Talks on Medical Electricity and Batteries, Bigelow.........447
Practical Intestinal Surgery, Robininson.........................620
Saunders' Pocket Medical Lexicon, Keating and Hamilton.......... 30
Saunders' Question Compends....619
Self-Examination for Medical Students.............................619

PAGE.
The Physician's Visiting List......29
The Medical Bulletin Visiting List for 1891............................31
The Medical News Visiting List for 1891..............................32
Twelve Lectures on the Structure of the Central Nervous System, Edinger..............................146
Text-Book of Hygiene, The Second Edition of the, Rohe...............146
The Year Book of Treatment for 1891..............................251
The Daughter, Her Health, Education and Wedlock, Capp........252
The Pocket Materia Medica and Therapeutics, Leonard ........396
Transactions of the Southern Surgical and Gynæcological Association.....................................448
Tables for Doctor and Druggist, Long..............................503
The Urine, The Common Poisons and the Milk, Holland................561
Weekly Medical Review Visiting List..............................32

### EDITORIAL.

Another Appeal......................35
A Request to our Readers............88
A Subject for thought.............305
Cremation.............................86
Congress of American Physicians and Surgeons......................249
Dr. E. V. Joye........................88
Dr. Benjamin Lee.....................154
Dr. Jas. A. Lydston.................154
Dr. Thos. S. Powell's Address to the Graduating Class, Southern Medical College.....................207
Dr. J. McF. Gaston.................304
Dr. Wm. Perrin Nicolson.........353
Dr. G. Frank Lydston..............402
Dr. J. B. S. Holmes.................402
Dr. J. B. S. Holmes' Sanitarium...449
Dr. Mark O'Daniel..................566
Dr. H. M. Whelpley.................566
Dr J. McF. Gaston..................617
Dr. A. G. Hobbs.....................617
Examining Board.....................449
Frequency of Diabetes.............249
Future of Marriage..................500
Georgia State Medical Association,194
Higher Medical Education.........352
International Congress of Hygiene and Demography...................352
Inter-Continental American Medical Congress......................402
J. B. Lippincott Co..................451
LaGrippe...............................36
Medicine in Georgia..................615
National Association of Railway Surgeons, Meeting of the........195
New Pork Polyclinic.................250
New York Medical Abstract...... 305
Philadelphia Polyclinic...............87
Practical Hints........................248
Prescription Department............353
Prescription Writing.................401
Rules for Bathing.....................250
Substitution............................450
Southern Associations...............498
The American Academy of Medicine...................................87
The Atmospheric Tractor............88
To Our Readers.........................89
The Medical Student and his Preceptor...............................148
Thirty-Third Annual Commencement of the Atlanta Medical College...............................150
Twelfth Annual Commencement of the Atlanta Medical College......150
To My Junior Brethren...............193
The State Medical Association....303
The North Carolina State Board of Examination........................357
The Woman's Medical College of Georgia..............................402
The Grady Hospital...................498
The Doctor's Bill......................499
The Future of Mosquitoes..........500
Tariffs Governing Railroad Surgeons..................................563
The Doctor Bill Vetoed.............565
The New York Journal of Gynæcology and Obstetrics............617
The Texas Sanitarium...............618

### SELECTIONS AND ABSTRACTS.

After-Pains........................... 34
Anæsthesia and Amenorrhœa.....56
Amenorrhœa and Dysmenorrhœa, a New Remedy in....................90
Acute Tonsilitis--Phagadenic Chancroid.................................99
Asthma..................................156
A Stimulating Expectorant.......186

PAGE.
Atropine in Diseases of the Heart, On The Action of................360
Administration of Iron, Some Notes Bearing upon the.. .....102
Arsenite of Copper in Diarrhœa..463
Amenorrhœa, Treatment of......464
A Birth of a Live Child at Six Months and a Half...............568
Alcohol and Digestion.............360
Bronchial Asthma..................95
Bandage After Labor,............253
Belladonna in Labor..............410
Broncho-Pneumonia in Children, Treatment of, by Sub-Cutaneous Injections of the Hydrochlorate of Quinine.......................621
Chapped Hands, Menthol for......23
Chapped Lips.........................46
Cardiac Hypertrophy............ .47
Counter Indications of Antipyrine.47
Chordee ....... ................ .85
Constipation in Infancy, Management of......... ... . ......98
Carbolic Acid Antidote.. .........101
Children Threatened with Croup.186
Chronic Bronchitis................204
Cephalhæmatoma and Teratomata.225
Convulsions in Children, Treatment of.............................253
Chronic Rheumatism......... ... 260
Chlorosis, Flint's Pill in. ...... .408
Corneal Affections, a New Method of Diagnosing Certain .. .......397
Closing Large Defects in the Intestines, a New Method of.........454
Cocaine Poisoning.................464
Creolin in Dysentery, Serous Diarrhœa and Summer Complaint ..511
Chancroids, the Dry Treatment of.543
Diarrhœa, Sub-Acute or Chronic..206
Dysmenorrhœa and Nervous Head ache, Pains of..................206
Diarrhœa of Puerperal Septicæmia ......... . .................... 260
Diagnosis Between Intra and Extra Capsular Fractures of Femur...302
Death after a Dose of Salol........350
Diagnosis of Head Injury from Drunkenness ........... .. .....567
Dressing of the Chest in Pneumonia Pleurisy, Pleurodynia, etc......622
Ergot in Obstetrical Practice, Its Use and Abuse ................182
Ear-Ache...:......................252
Entire Excision of the Tongue ...505
Fissured Nipple, Treatment of.... 96
Fetid Breath, Pastils for..........259
Felons, Treatment of... ........305
Faridization in Incontinence of Urine ...........................356
Fistula in Ano................. ...462

PAGE.
Gonorrhœal Rheumatism. ........44
Gonorrhœa ........ ............. .95
Germicidal Action of the Blood, The...........................155
Gluten as a Food...................199
Good on the Belly Rippers .....410
Gunshot Wound of the Pregnant Uterus......................... 92
Headache of Children.............93
Harrassing Cough ................182
Hydrogen Dioxide—a Resume....256
Hemorrhage........................461
Headaches, The Treatment of....513
Iritis, Dr. A. W. Calhoun's Treatment for......... ............. 56
Infantile Convulsions.............147
Inflammation of the Breast Simulating Cancer.....................101
Infantile Convulsions.............147
Iodide of Potassium in Treatment of Urticaria................... 260
Iodoform in Chronic Eye Diseases.356
Infantile Diarrhœa............... 462
Ingrown Toe Nail, Simple Treat ment of........................ 359
Industrial Future of the South, The ...................................47
LaGrippe............ ... .........97
Leucorrhœa.......... ....... ....260
Lavage of the Stomach in Young Children..........................618
Mosquera's Food Products—Beef Meal, Beef Cacao ............... 40
Menstruation, a New Theory of...407
Malt Extract—a Therapeutic Study ............... ...... ........ 506
Neuralgic Spermatica, Treatment of.............................. 82
Neuropathology ...................453
Neurosymptomatology............457
Nicotine on the Circulation, The Action of........ .. · .........458
Neuralgia Following Fracture, Successfully Treated by Operation..459
On Exalgine as an Analgesic......254
Peritonitis..........................19
Pneumonia, The Rational Treatment of.................... ......45
Painless Circumcision.......... ....94
Pneumonia in Very Young Infants, The Diagnosis and Treatment of ................................. 198
Pneumonia...........................204
Pruritis Ani..... . ................310
Permanganate of Potassium in Diphtheria..........................409
Perioxide of Hydrogen for Cleaning the Hands.......... ........412
Pelvic Inflammation and the General Practitioner............... 455

Prevention of R t on Surgical Needles....... ........,........566
Pilocarpine in Puerperal Eclampsia ..........................562
Periods of Gestation..............562
Rickets, Treatment of........100, 254
"Red Nose," Treatment of........307
Rectal Obstruction in a Child, Case of.............................. 82
Suicides in France.................23
Somnal, the New Hypnotic, Notes upon............................ 37
Swallowed a Gold Safety pin...... 55
Simple Pneumatic Method of Removing Foreign Substances from Nasal Cavity of Children......... 93
Soft Chancres, Treatment of....... 97
Salol in Typhoid Fever............156
Sub-acute Pleurisy................186
Some New Pharmaceutical Preparations, Clinical Observations on 205
Solutions of Corrosive Sublimate, the use of......................255
Summer Disturbances of Children 298
Self Adjusting Pneumatic, The....362
Syphilis not of American Origin...409
Tonsilitis......................... 48
Tonsilitis, Antiseptic Treatment of 48
Tetany in Children............... 97
Three Double Births..............100
Therapeutic Value of Salicyl-Bromanalid, Some Experimental and Clinical Observations upon the..403
Treatment of Boils................410
Trephining, Indications for.......412
Unconscious Parturition in a Primapara............................196
Vomiting in Pregnancy............491
Varicose Ulcers, The Treatment of 309
Vomiting of Pregnancy, Elevation of the Pelvis as a means of Relieving............................411
Varicocele, The Treatment of.....463
Washing out the male Bladder with out the use of a Catheter, a Proceedure for...................... 98
Whooping Cough, Treatment of...259
Water in Typhoid Fever, Internal use of .........................408

### PRESCRIPTION DEPARTMENT.

Amenorrhœa.......................363
Aid to Digestion..................416
Acute Cystitis....................467
Acute Bronchitis..................468
Amenorrhœa.......................519
Acute Cold........................573
Asthma, Fumigant for.............415
Acute Laryngitis, For.............572
Ballanitis, Treatment of..........415
Bronchitis........................626
Burns and Scalds.................467
Consumption......................313
Chronic Cystitis in Women.......314
Constipation and Flatulency......364
Cough Mixture....................415
Chronic Gonorrhœa............. 415
Cholera Morbus...................467
Cholera Infantum.................468
Constipation......................626
Corns.............................468
Convulsions.......................626
Diarrhœa Mixture.................313
Dental Caries.....................363
Debility of Grippe................416
Difficult Digestion, Accompanied with Constipation and Flatulency................................520
Diarrhœa......................... 520
Dental Caries, To Prevent........520
Erysipelas........................363
Ear-Ache Drops...................415
Eczema...........................416
Eczema of Bones and Scrotum....519
Eczema and Psoriasis.............627
Fissured Nipples..................363
Fetid Breath, For.................520
Flatulence........................573
Gastric Catarrh...................313
Hemorrhoids, Ointment for.......313
Hemorrhoids......................314
Herpes............................514
Heart Stimulant...................467
Itch, Ointment for............... 416
Irritable Heart....................571
Neuralgia and Rheumatism ......572
Pigmentations in Pregnant Women, Treatment of.....................415
Pediculus Pubis...................416
Prolapsus Recti...................467
Profuse Purulent Expectoration..573
Pleurisy......................... 626
Pneumonia........................626
Rigid Perinæum.................. 313
Ringworm.........................314
Rossback's Expectorant Mixture..572
Rheumatism......................573
Summer Diarrhœa.................363
Stomatitis in Children............519
Spasmodic Cough.................519
Tonsilitis......................314–416
Varicose Veins....................468
Whooping Cough...............313–416
Warts.............................314

